# Intraocular Cytokine Level Prediction from Fundus Images and Optical Coherence Tomography

**DOI:** 10.3390/s25237382

**Published:** 2025-12-04

**Authors:** Hidenori Takahashi, Taiki Tsuge, Yusuke Kondo, Yasuo Yanagi, Satoru Inoda, Shohei Morikawa, Yuki Senoo, Toshikatsu Kaburaki, Tetsuro Oshika, Toshihiko Yamasaki

**Affiliations:** 1 Center for Cyber Medicine Research, University of Tsukuba, Tsukuba 305-8575, Japan; 2 Department of Ophthalmology, Jichi Medical University, Shimotsuke 329-0498, Japan; r1208is@jichi.ac.jp (S.I.); kabutosi@jichi.ac.jp (T.K.); 3 DeepEyeVision Inc., Shimotsuke 329-0498, Japan; t-tsuge@deepeyevision.com (T.T.); y-kondo@deepeyevision.com (Y.K.); 4 Department of Visual Reconstructive Surgery, Yokohama City University, Yokohama 232-0024, Japan; yanagi.yas.wu@yokohama-cu.ac.jp; 5 Retina Research Group, Singapore Eye Research Institute, Singapore 168751, Singapore; 6 Department of Ophthalmology, University of Tsukuba, Tsukuba 305-8575, Japan; shomorikawa@md.tsukuba.ac.jp (S.M.); oshika@eye.ac (T.O.); 7 Department of Ophthalmology, Nihon University, Tokyo 173-8610, Japan; senooyuki0821@gmail.com; 8 Department of Technology, the University of Tokyo, Tokyo 113-8656, Japan; yamasaki@cvm.t.u-tokyo.ac.jp

**Keywords:** deep-learning, cytokine, optical coherence tomography

## Abstract

**Highlights:**

**What are the main findings?**

- Deep learning models using fundus and OCT images showed limited ability to predict intraocular cytokine concentrations, with overall low R^2^ values across approaches.
- Including demographic and clinical features did not improve model performance compared with image-only inputs.

**What is the implication of the main finding?**

- The findings suggest that cytokine levels may not be strongly reflected in retinal image features alone.
- Further refinement of model design and incorporation of additional modalities may be needed to achieve reliable prediction.

**Abstract:**

The relationship between retinal images and intraocular cytokine profiles remains largely unexplored, and no prior work has systematically compared fundus- and OCT-based deep learning models for cytokine prediction. We aimed to predict intraocular cytokine concentrations using color fundus photographs (CFP) and retinal optical coherence tomography (OCT) with deep learning. Our pipeline consisted of image preprocessing, convolutional neural network–based feature extraction, and regression modeling for each cytokine. Deep learning was implemented using AutoGluon, which automatically explored multiple architectures and converged on ResNet18, reflecting the small dataset size. Four approaches were tested: (1) CFP alone, (2) CFP plus demographic/clinical features, (3) OCT alone, and (4) OCT plus these features. Prediction performance was defined as the mean coefficient of determination (R^2^) across 34 cytokines, and differences were evaluated using paired two-tailed *t*-tests. We used data from 139 patients (152 eyes) and 176 aqueous humor samples. The cohort consisted of 85 males (61%) with a mean age of 73 (SD 9.8). Diseases included 64 exudative age-related macular degeneration, 29 brolucizumab-associated endophthalmitis, 19 cataract surgeries, 15 retinal vein occlusion, and 8 diabetic macular edema. Prediction performance was generally poor, with mean R^2^ values below zero across all approaches. The CFP-only model (–0.19) outperformed CFP plus demographics (–24.1; *p* = 0.0373), and the OCT-only model (–0.18) outperformed OCT plus demographics (–14.7; *p* = 0.0080). No significant difference was observed between CFP and OCT (*p* = 0.9281). Notably, VEGF showed low predictability (31st with CFP, 12th with OCT).

## 1. Introduction

Intraocular cytokine concentrations play a crucial role in ocular pathology [1,2,3,4]. Intraocular cytokine concentrations play a crucial role in ocular pathology [1,2,3,4]. Anti-VEGF therapy, which has substantially reduced the global burden of blindness [5], was originally developed following studies that demonstrated correlations between intraocular VEGF levels and disease severity [1,2,3]. Numerous investigations have since examined associations between intraocular protein concentrations and diverse ocular disorders, thereby advancing our understanding of disease mechanisms. For example, elevated intraocular PDGF levels observed in proliferative diabetic retinopathy have highlighted the importance of PDGF in fibrovascular proliferation [4]. Anti-VEGF therapy, which has substantially reduced the global burden of blindness [5], was originally developed following studies that demonstrated correlations between intraocular VEGF levels and disease severity [1,2,3]. Numerous investigations have since examined associations between intraocular protein concentrations and diverse ocular disorders, thereby advancing our understanding of disease mechanisms. For example, elevated intraocular PDGF levels observed in proliferative diabetic retinopathy have highlighted the importance of PDGF in fibrovascular proliferation [4].

However, obtaining aqueous humor samples in routine clinical practice is inherently invasive and therefore restricted to circumstances with clear clinical justification, such as diagnostic evaluation for intraocular lymphoma [6]. Globally, aqueous humor sampling for cytokine measurement has remained largely confined to research settings or performed incidentally during other intraocular procedures. Consequently, most published studies have been limited to relatively small cohorts and have relied on conventional statistics or, at most, relatively simple machine learning approaches such as random forests [7,8]. Given the high dimensionality of cytokine profiling data, hundreds of cases are generally required to train more advanced algorithms, a scale that is practically unattainable in a single-center prospective study. These constraints have hindered the translation of cytokine profiling into practical clinical tools and have left unexplored whether more powerful machine learning algorithms applied to larger integrated datasets could yield deeper insights or enable clinically useful prediction.

Recent advances in artificial intelligence have demonstrated that deep learning models can infer systemic or molecular states directly from non-invasive imaging and clinical data, suggesting a broader potential for image-based biomarker prediction. For example, large-scale multimodal learning frameworks have successfully estimated metabolic and inflammatory status from retinal photographs [9], while data-driven feature extraction approaches have been shown to capture molecular signatures from structural medical images [10]. In addition, multimodal models integrating imaging and clinical profiles have improved the prediction of systemic biomarkers such as HbA1c and cardiometabolic risk factors [11]. These developments indicate that modern machine learning can recover biological information beyond human-perceptible image features, raising the question of whether retinal imaging may likewise contain sufficient signal to approximate intraocular cytokine concentrations.

Building on these considerations, we aimed to develop machine learning models to predict intraocular cytokine concentrations from retinal imaging modalities such as color fundus photographs (CFP) and optical coherence tomography (OCT). Over the past decade, we have collected more than 20,000 intraocular fluid samples. From these, we analyzed the cytokine concentrations of specimens that had both CFP and OCT examinations performed within one week of each other, using machine learning techniques. The cytokine measurement results of these samples have been individually reported over the past ten years in disease-specific analyses using conventional statistical approaches such as multivariate regression [12,13,14,15,16,17,18,19,20,21]. These previous studies originally established the detailed inclusion criteria and cytokine measurement protocols. These references are self-citations but are indispensable for ensuring methodological transparency and data provenance. By analyzing the dataset without separating it by disease category, however, we hypothesized that advanced machine learning approaches might enable more comprehensive inference and that cytokine profiles may serve as biological intermediates linking retinal images with clinical phenotypes.

We consider cytokine profiles as potential biological intermediates between retinal imaging and clinical phenotypes. In this sense, cytokine inference from imaging does not merely aim to replace invasive sampling, but rather represents a conceptual step toward future AI systems that can capture molecular and cellular states beyond expert human interpretation of images. We therefore regarded the present study as an exploratory attempt to test whether large-scale integration of cytokine datasets could support machine learning models capable of approximating intraocular cytokine profiles. Such efforts may ultimately contribute to the development of next-generation AI frameworks that integrate structural imaging with biological context.

The contributions of this study are threefold.

First, we present the largest integrated dataset to date combining retinal imaging (CFP and OCT) with intraocular cytokine measurements, enabling analysis beyond the scope of conventional disease-specific studies.

Second, we systematically compare multiple deep learning pipelines for cytokine prediction, incorporating image-only, multimodal, and clinical feature–augmented models, thereby clarifying the extent to which current algorithms can extract cytokine-related information from retinal images.

Third, by evaluating model behavior across 34 cytokines—including those of high clinical relevance such as VEGF—we provide new empirical evidence regarding the feasibility and limitations of inferring intraocular molecular states from noninvasive imaging. These findings help define realistic boundaries for future AI systems aiming to integrate structural imaging with underlying biological context.

The remainder of this paper is organized as follows. Section 2 describes the dataset, cytokine measurement procedures, and imaging modalities. Section 3 presents the results of the prediction experiments. Section 4 discusses the implications, limitations, and potential applications of cytokine inference from retinal imaging. Section 5 concludes the study.

## 2. Materials and Methods

### 2.1. Study Design and Approval

This study was conducted under the ethical approvals of both Jichi Medical University (E12-86) and the Japan Community Healthcare Organization Tokyo Shinjuku Medical Center (H22-8-9), in accordance with the Declaration of Helsinki (2013 revision). All participants provided written informed consent, and the study was registered with the University Hospital Medical Information Network (UMIN000020718).

The present cohort comprised eyes with a variety of retinal diseases as well as control eyes. Whereas conventional studies often restrict their analyses to single disease groups (e.g., comparing AMD with normal controls), deep learning models are generally better trained on more heterogeneous case distributions to reduce bias and improve generalizability. Therefore, we chose not to restrict the analysis to a single disease category.

### 2.2. Outcome Variazbles

The details of sample collection procedures and multiplex cytokine assays have been described previously [12,13,14,15,16,17,18,19,20,21]. For cytokines that were measured, values below the detection limit were replaced with one-half of the minimum detectable concentration in order to preserve the log-normal distribution generally observed in cytokine levels. Because cytokine concentrations typically follow a log-normal distribution, all cytokine values were log^10^-transformed prior to model training and statistical analysis. Because different studies measured different sets of cytokines, the presence or absence of values depended on which cytokines the investigators had chosen to analyze in each study. These missing values were therefore considered to be missing at random (MAR). Missing values not measured, regarded as MAR, were imputed using the Automated Data Imputation (ADI) method implemented in JMP Pro 18.0.2 (JMP Statistical Discovery LLC, Cary, NC, USA). The ADI method imputes missing values by applying a low-rank matrix approximation based on the Soft-Impute algorithm, which automatically determines the optimal rank from the data and provides a flexible and robust approach applicable to various types of datasets. In total, cytokine concentrations were available for 825 aqueous humor samples across the ten studies, and these were used as the basis for data imputation.

### 2.3. Predictor Variables

**Figure 1:**
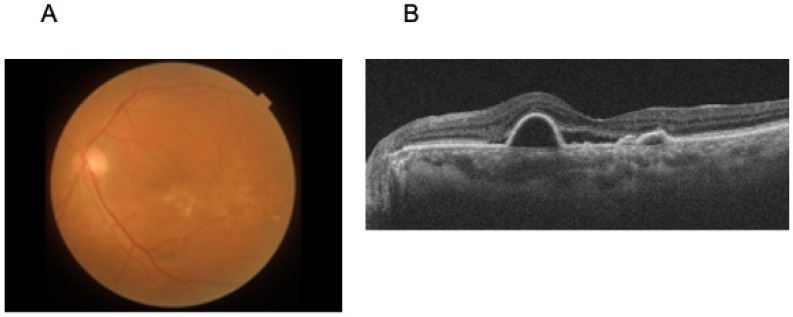
Representative input images used in the analysis. (**A**) Color fundus photograph (45° central field) from a 76-year-old female patient with exudative age-related macular degeneration, (**B**) Horizontal macular B-scan OCT from the same eye, showing the central foveal region. These examples illustrate the imaging modalities used as inputs for model construction.

CFP and OCT were obtained within one week of aqueous humor sampling. For CFP, only 45° central color fundus photographs were used, and for OCT, only central horizontal B-scan images were included in the analysis. In addition, demographic and clinical information was extracted from medical records, including age, sex, axial length, best-corrected visual acuity, history of prior treatment, time since prior treatment, history of cataract surgery, history of vitrectomy, posterior vitreous detachment status, and central retinal thickness. Among these, 176 samples had both CFP and OCT images available and were included in the present analysis. OCT images were supplied to the AutoGluon framework without manual resizing. AutoGluon automatically applied its standard preprocessing pipeline, which includes resizing and center-cropping each B-scan to match the input dimensions required for pretrained convolutional neural networks (224 × 224 pixels). This procedure ensures compatibility with CNN-based feature extraction while preserving the essential macular structures within the central slice. Representative examples of the CFP and OCT images used in the analysis are shown in Figure 1A,B. Of the 35 cytokines measured, Galectin-1 was excluded because only two samples had observed values, resulting in unstable estimates.

### 2.4. Model Construction

**Figure 2:**
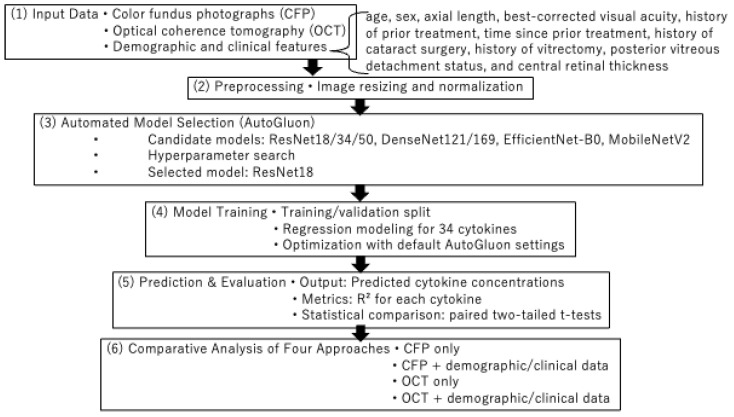
An overview of the analytical workflow. This flowchart summarizes the sequential steps of the proposed algorithm, including (1) input data (CFP, OCT, and demographic/clinical features), (2) preprocessing with automated image resizing and normalization, (3) model selection within AutoGluon including exploration of multiple CNN architectures (converging on ResNet18), (4) model training for 34 cytokines, (5) prediction and evaluation using R^2^ and paired *t*-tests, and (6) comparison of the four analytical approaches.

An overview of the analytical workflow is summarized in Figure 2. We employed the AutoGluon library (stable release as of May 2025; https://auto.gluon.ai/, accessed on 18 August 2025) implemented in Python 3.10 on Ubuntu Linux (LTS release). AutoGluon automatically selects and optimizes machine learning algorithms such as CatBoost and LightGBM for structured data and applies convolutional neural networks such as ResNet and DenseNet for image data. OCT images were fed into the AutoGluon framework without manual resizing; AutoGluon automatically applied its standard image preprocessing pipeline (resizing and center-cropping to 224 × 224 pixels) before extracting latent embeddings with convolutional neural networks (e.g., ResNet18). AutoGluon automatically integrates image and structured modalities by extracting latent embeddings from CNNs (e.g., ResNet18) for CFP and OCT images, and combining them with demographic/clinical variables modeled using algorithms such as CatBoost and LightGBM. These components are fused through a stack-ensemble architecture, in which AutoGluon internally explores multiple multimodal combinations and selects the best-performing model through cross-validation. Thus, all demographic and clinical features were incorporated into the fusion process without manual tuning. Demographic and clinical variables were processed using AutoGluon’s default tabular preprocessing pipeline, which performs categorical encoding, and model-appropriate handling of numerical variables. No additional manual normalization was applied, as the tree-based models (LightGBM, CatBoost) used for tabular features do not require feature scaling. Prediction models were trained using these methods, and cytokines were subsequently ranked according to their prediction accuracy, as evaluated by the coefficient of determination (R^2^). To assess the contribution of different input modalities, four approaches were tested: (1) CFP alone, (2) CFP combined with demographic and clinical features, (3) OCT alone, and (4) OCT combined with these features.

### 2.5. Statistical Analysis

Prediction performance was defined as the mean coefficient of determination (R^2^) across the 34 cytokines. Differences in prediction accuracy between models were evaluated using paired two-tailed Student’s *t*-tests conducted in JMP Pro 18.0.2 (JMP Statistical Discovery LLC, Cary, NC, USA). A significance level of α = 0.05 was adopted.

## 3. Results

### 3.1. Patient Characteristics

A total of 176 aqueous humor samples were collected from 152 eyes of 139 patients across multiple multiplex cytokine assay studies. The cohort consisted of 85 males (61%) and 54 females, with a mean age of 73 years (SD, 9.8). The underlying diseases included exudative age-related macular degeneration (*n* = 64), brolucizumab-associated intraocular inflammation (*n* = 29), cataract surgery controls (*n* = 19), retinal vein occlusion (*n* = 15), diabetic macular edema (*n* = 8), tilted disc syndrome (*n* = 1), and macular pucker (*n* = 1) (Table 1). Figure 2 shows a typical example of CFP and OCT, along with age, sex, and cytokine measurement values.

### 3.2. Image Acquisition

Fundus images were obtained using Triton (Topcon Corporation, Tokyo, Japan; *n* = 94), VX-10 (Kowa Company, Ltd., Tokyo, Japan; *n* = 52), and other devices including Optos (Optos plc, Dunfermline, United Kingdom) and VX-20 (Kowa Company, Ltd., Tokyo, Japan). OCT images were available for 157 eyes. Among these, 111 images were captured with Triton (Topcon Corporation, Tokyo, Japan), while the remainder were taken with Atlantis (Topcon Corporation, Tokyo, Japan) or RS-3000 (Nidek Co., Ltd., Gamagori, Japan).

### 3.3. Cytokine Prediction Using CFP

The CFP-only model demonstrated significantly better performance than the model incorporating demographic and clinical variables (mean R^2^ –0.19 vs. –24.1; *p* = 0.0373). Based on the CFP-only model, the cytokines with the highest R^2^ values, indicating the highest prediction accuracy, were IL-17A, IL-8, and MCP-1. In contrast, the cytokines with the lowest R^2^ values, reflecting the lowest prediction accuracy, were G-CSF, MMP-9, and IL-12 p70. Notably, VEGF ranked 30th out of the 34 cytokines in terms of prediction accuracy (Table 2).

### 3.4. Cytokine Prediction Using OCT

**Figure 3:**
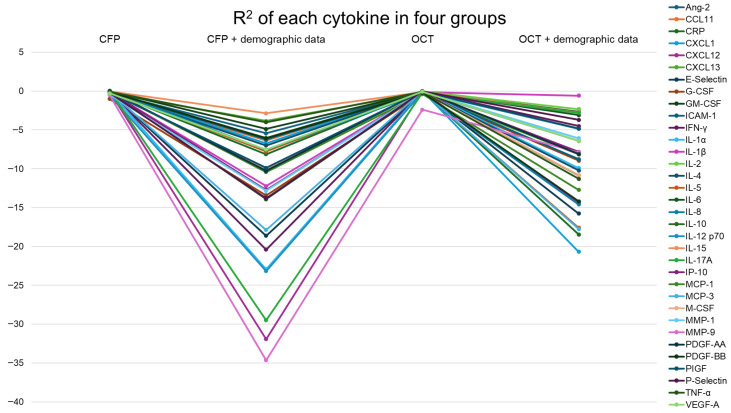
Coefficient of determination (R^2^) for each cytokine across the four analysis groups. CFP alone, CFP plus demographic/clinical features, OCT alone, and OCT plus these features. Bars represent prediction performance for 34 cytokines.

Similarly, the OCT-only model outperformed the OCT plus demographics model (mean R^2^ –0.18 vs. –14.7; *p* = 0.0080). In the OCT-only analysis, two cytokines demonstrated positive R^2^ values. The top three cytokines with the highest R^2^ were PDGF-AA, MCP-3, and CXCL1, whereas the lowest were MMP-9, MCP-1, and G-CSF. VEGF ranked 12th among the 34 cytokines (Table 2, Figure 3). Notably, the predictive performance remained stable even when plotted against the number of observed (not imputed) samples for each cytokine, indicating that the results were not substantially affected by the variation in sample size (see Figure S1). An illustrative example of predicted versus observed IL-6 concentrations is provided in Figure S2.

### 3.5. Comparison Between CFP and OCT

**Figure 4:**
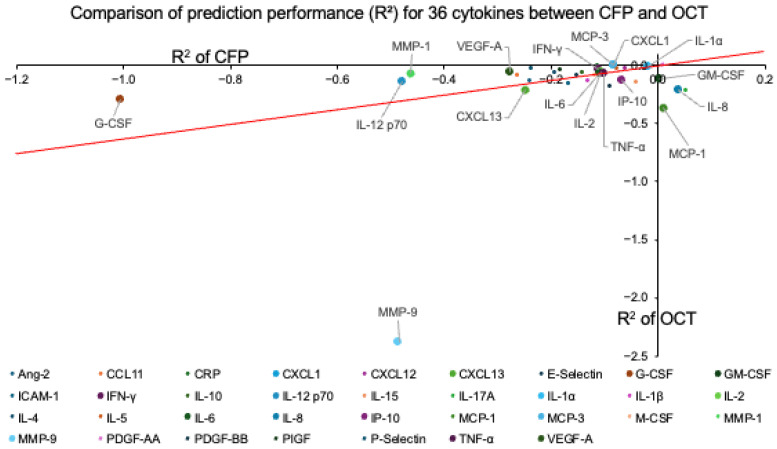
Comparison of prediction performance (R^2^) between CFP- and OCT-based analyses for 34 cytokines. Each point represents one cytokine, plotted using R^2^ values obtained from CFP-based predictions (x-axis) and OCT-based predictions (y-axis). The red line represents the regression line fitted across cytokines.

When the CFP-only and OCT-only models were directly compared, no significant difference in overall performance was observed between the two modalities (*p* = 0.9281). To further explore differences at the individual cytokine level, the R^2^ values of OCT minus CFP were calculated for each cytokine. The top three cytokines with the largest positive differences were G-CSF, MMP-1, and IL-12 p70, whereas the largest negative differences were MMP-9, MCP-1, and IL-17A. Notably, VEGF ranked 4th among the 34 cytokines in this comparison. For clarity, Figure 4 illustrates the comparison as a scatter plot, with selected cytokines labeled to highlight representative examples.

(OCT R^2^ = –0.062053 + 0.6495 × CFP R^2^),

illustrating the overall relationship between the two imaging modalities. To reduce visual clutter while emphasizing cytokines supported by more robust empirical data, labels are shown only for cytokines with 150 or more directly measured (non-imputed) samples. Negative R^2^ values indicate poor predictive performance relative to a mean predictor.

## 4. Discussion

Most previous studies have compared cytokine concentrations among several groups [12,13,14,16,17,18,20,21,22,23,24], and some have used cytokine levels as explanatory variables to predict disease diagnosis or prognosis. These analyses have primarily relied on multivariate statistical models [15,19], with only a few reports applying machine learning approaches [7,8]. These approaches, however, provide only indirect estimations based on clinical classifications rather than image-derived features. In contrast, the present study attempted to estimate aqueous humor cytokine concentrations directly from ocular imaging, specifically CFP and OCT, thereby offering a more granular and potentially pathophysiologically relevant approach.

However, previous machine learning studies were limited by (1) small disease-specific datasets, (2) restricted cytokine panels, and (3) models that depended on manually selected clinical variables, which may not fully capture the morphological information embedded in retinal images. These constraints prevented those methods from assessing whether imaging contains sufficient signal to approximate intraocular molecular states. By integrating a larger cytokine dataset and systematically examining image-only and multimodal deep learning pipelines, our study provides a clearer evaluation of these methodological limitations and demonstrates both the potential advantages and the remaining challenges of cytokine prediction from retinal imaging.

Our findings yielded several noteworthy observations. First, adding demographic or clinical information did not improve prediction accuracy, suggesting that image-based features alone contained the dominant signals relevant to cytokine profiles. Second, OCT achieved higher accuracy than CFP, particularly in eyes with age-related macular degeneration (AMD). This indicates that OCT may better capture morphological correlates of intraocular cytokine activity compared with CFP, which provides only en face retinal images. Interestingly, the prediction accuracy for VEGF, the most clinically relevant cytokine in neovascular AMD, was modest, highlighting that not all cytokines can be equally inferred from structural imaging features.

These results have several implications. They suggest that intraocular cytokine estimation from OCT could serve as an intermediate surrogate linking noninvasive imaging to intraocular molecular pathology. Such surrogate markers may help in understanding disease mechanisms, stratifying patients, and evaluating treatment response, particularly in conditions such as AMD where cytokine signaling plays a pivotal role. Furthermore, the observation that demographics did not meaningfully enhance performance underscores the central importance of image-derived information in modeling intraocular biology.

Notably, VEGF ranked substantially higher when comparing OCT with CFP, reaching fifth place in the OCT–CFP differential analysis. A plausible explanation is that VEGF-driven edema [25] is more readily visualized on OCT than on CFP. This finding suggests that further investigations may help clarify which cytokines are preferentially reflected in specific imaging features, thereby supporting the value of pursuing cytokine-specific imaging correlates.

### Limitations

This study has several limitations. First, all participants were Japanese, and the findings may not generalize to populations with different ethnic backgrounds or fundus pigmentation. Second, the cohort was skewed toward age-related macular degeneration, reflecting the authors’ specialty and the patient population of a tertiary referral center. Although this reflects real-world practice in such settings, the distribution is not representative of the general population. Third, the overall sample size was modest, which may have limited the prediction accuracy of the deep learning models. Larger, multi-center studies with more balanced case distributions are needed to confirm and extend these findings. In addition, while OCT outperformed CFP in this study, future work should explore whether multimodal imaging or more advanced model architectures can improve prediction of difficult targets such as VEGF.

## 5. Conclusions

Overall prediction performance was limited, with mean R^2^ values below zero across all models (e.g., –0.19 for CFP-only and –0.18 for OCT-only). Demographic and clinical factors did not meaningfully improve prediction accuracy. Current deep learning approaches appear insufficient for reliable cytokine prediction from imaging alone. Future work should involve larger multi-institutional datasets, more advanced architectures, and multimodal integration to better capture intraocular molecular states.

## Figures and Tables

**Table 1:** Participant Characteristics. Summary of demographic and clinical data of the study participants.

Variable	Value
Number of aqueous humor samples	176 from 152 eyes of 139 patients
Number of participants	Male: 85, Female: 54
Age (years)	Mean: 73, SD: 9.8, Range: 36–101
Exudative Age-related Macular Degeneration	64
Brolucizumab-associated Intraocular Inflammation	29
Cataract Surgery as Controls	19
Retinal Vein Occlusion	15
Diabetic Macular Edema	8
Tilted Disc Syndrome	1
Macular Pucker	1

**Table 2:** The Coefficient of Determination (R^2^) of each Cytokine Concentrations. Coefficient of determination values for each of the 34 cytokines under four prediction settings: color fundus photography (CFP), CFP combined with demographic features, optical coherence tomography (OCT), and OCT combined with demographic features. The number of observed samples used for each cytokine is also shown.

Cytokine	CFP	CFP & Demographic Features	OCT	OCT & Demographic Features	Number of Observed Samples
Ang-2	−0.24	−5.4	−0.14	−6.4	22
CCL11	−0.26	−6.4	−0.097	−7.9	31
CRP	−0.11	−8.1	−0.0057	−18	22
CXCL1	−0.082	−6.8	−0.0028	−21	154
CXCL12	−0.059	−32	−0.038	−14	50
CXCL13	−0.25	−3.8	−0.21	−8.2	176
E-Selectin	−0.15	−19	−0.09	−16	101
G-CSF	−1	−13	−0.3	−9	150
GM-CSF	0.0023	−4.9	−0.12	−2.7	150
ICAM−1	−0.17	−6.2	−0.16	−8.8	75
IFN-γ	−0.11	−20	−0.039	−4.5	150
IL-1α	−0.018	−23	−0.0081	−9.9	150
IL-1β	−0.13	−12	−0.15	−0.6	101
IL-2	−0.11	−10	−0.056	−2.3	150
IL-4	−0.19	−7	−0.064	−10	75
IL-5	−0.075	−7.8	−0.034	−18	101
IL-6	−0.11	−14	−0.064	−3.1	176
IL-8	0.038	−7.6	−0.21	−15	172
IL-10	−0.14	−6	−0.071	−11	105
IL-12 p70	−0.48	−23	−0.14	−4.8	150
IL-15	−0.039	−2.9	−0.15	−2.7	49
IL-17A	0.053	−29	−0.22	−2.8	75
IP-10	−0.067	−13	−0.13	−7.8	176
M-CSF	−0.12	−10	−0.096	−11	53
MCP-1	0.011	−10	−0.37	−13	176
MCP-3	−0.083	−18	0.00009	−18	150
MMP-1	−0.46	−13	−0.067	−6.1	150
MMP-9	−0.49	−35	−2.4	−7.9	150
P-Selectin	−0.24	−14	−0.033	−3.7	63
PDGF-AA	0.0092	−10	0.00022	−8.1	49
PDGF-BB	−0.088	−6.1	−0.18	−14	22
PIGF	−0.18	−9.9	−0.049	−4.8	22
TNF-α	−0.1	−4	−0.068	−11	150
VEGF-A	−0.28	−7.5	−0.062	−6.4	172

## Data Availability

All data and figures are available from the figshare database (https://doi.org/10.6084/m9.figshare.30154360.v1).

## Supplementary Materials

The following supporting information can be downloaded at: https://www.mdpi.com/article/10.3390/s25237382/s1, Figure S1: Comparison of predictions by number of observation samples and type of image; Figure S2: Scatter plot of observed vs. predicted IL-6 concentrations (log_10_ scale).

## Abbreviations

The following abbreviations are used in this manuscript:

CFP	Color fundus photographs
OCT	Optical coherence tomography
AMD	Age-related Macular Degeneration
MAR	Missing at random
ADI	Automated Data Imputation
