# Supplementary material for: Intraocular Cytokine Level Prediction from Fundus Images and Optical Coherence Tomography
Source: Sensors (Basel). 2025 Dec 4;25(23):7382. doi: 10.3390/s25237382 (PMC12694473; doi:10.3390/s25237382)
Supplement: Supplementary file 1 [file sensors-25-07382-s001.zip › sensors-3960509-supplementary.pdf]

sensors

Article

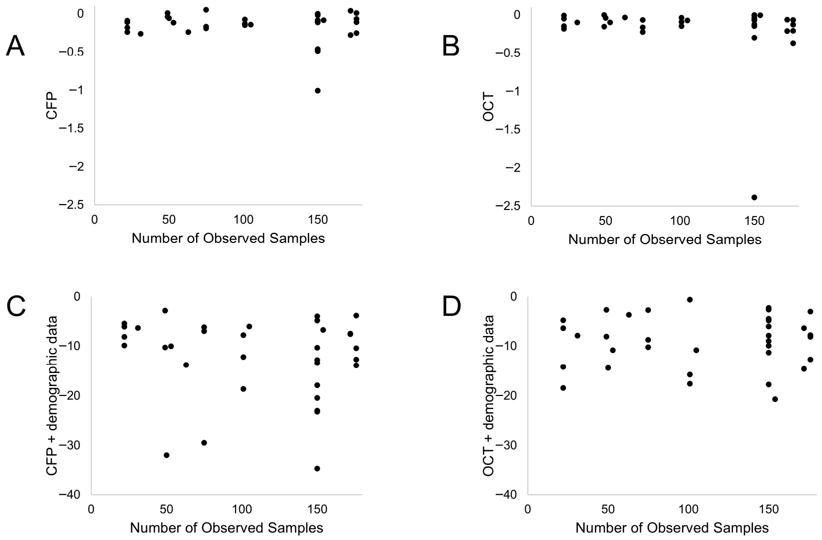

Figure S1. Comparison of predictions by number of observation samples and type of image. Scatter plot comparing the coefficient of determination (R^2) of cytokine concentration predictions obtained from CFP-based analyses (x-axis) and OCT-based analyses (y-axis). Each point represents one cytokine (n = 34). The red diagonal line represents the identity line (y = x), where points above the line indicate cytokines better predicted from OCT and points below indicate cytokines better predicted from CFP. To maintain readability, labels were added only for selected representative cytokines with relatively fewer missing values.

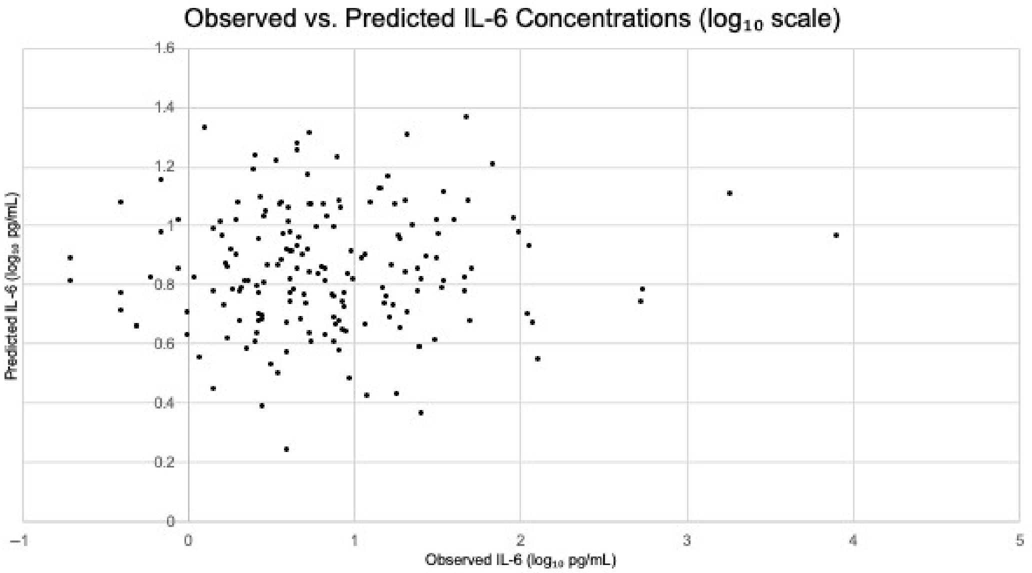

Figure S2. Scatter plot of observed vs. predicted IL-6 concentrations ($\log_{10}$ scale). Scatter plot showing the relationship between the observed IL-6 concentrations (x-axis, $\log_{10}$-transformed) and the predicted values generated by the machine-learning model (y-axis, $\log_{10}$-transformed). A substantial dispersion is visible, with predictions deviating widely from the measured values, including several predicted values extending into negative ranges despite physiologically positive IL-6 concentrations. This pattern reflects the low predictive performance for IL-6 in this study (R^2 = –0.064). The gray diagonal line represents the line of identity (y = x) for reference.

 https://doi.org/10.3390/s25237382
